# Maternal near miss and maternal deaths in Mozambique: a cross-sectional, region-wide study of 635 consecutive cases assisted in health facilities of Maputo province

**DOI:** 10.1186/s12884-014-0401-3

**Published:** 2014-12-10

**Authors:** Ernestina David, Fernanda Machungo, Giovanni Zanconato, Elena Cavaliere, Sidonia Fiosse, Celeste Sululu, Benvinda Chiluvane, Staffan Bergström

**Affiliations:** Department of Obstetrics and Gynecology, Maputo Central Hospital, Maputo, Mozambique; Department of Life Science and Reproduction, University of Verona, U.O. di Ginecologia e Ostetricia, Policlinico Borgo Roma, 37134 Verona, Italy; Division of Global Health, Karolinska Institutet, Stockholm, Sweden

**Keywords:** Near miss, Severe acute maternal morbidity, Maternal mortality, Substandard care

## Abstract

**Background:**

Life-threatening events during pregnancy are currently used as a measure to assess quality of obstetric care. The aim of this study is to assess prevalence of near miss cases and maternal deaths, to elucidate the causes and to analyze avoidable factors based upon the three-delays approach in southern Mozambique.

**Methods:**

Near miss cases comprised five categories: eclampsia, severe hemorrhage, severe sepsis, uterine rupture and severe malaria. Pregnant women surviving the event were interviewed during a 5-month period within five health facilities offering comprehensive emergency obstetric care in Maputo City and Province. Family members gave additional information and were interviewed in case of the patient’s death.

**Results:**

Out of 27,916 live births, 564 near miss cases and 71 maternal deaths were identified, giving a total maternal near miss ratio of 20/1,000 live births and maternal mortality ratio of 254/100,000 live births, respectively. Near miss fatality rate was 11.2%. Among near miss cases hemorrhage accounted for the most common event (58.0%), followed by eclampsia (35.5%); HIV seroprevalence was 22.3%. Inappropriate attendance in antenatal care services (21.1%), late or wrong diagnosis (12.6%), inadequate management immediately after delivery (9.6%), no monitoring of blood pressure and other vital signs (9.2%) were the most prevalent factors contributing to the severe morbidity under study. Third delay was identified in 69.7% of the interviews. In more than one fourth of near miss cases treatment was not started immediately. Lack of blood derivates and unavailable operating room were reported in 42.0% and 35.0%, respectively.

**Conclusions:**

Near miss cases were frequent and related to delays in reaching and receiving adequate care. First and third type of delay contributed significantly to the number of maternal near miss cases and deaths. Maternal health policies need to be concerned not only with averting the loss of life, but also with ameliorating care of severe maternal complications at all levels including primary care. Sexual and reproductive health services for adolescents should be prioritized to prevent adverse outcomes.

## Background

A near miss case has been defined any severe obstetric complication threatening the woman's immediate survival [1,2]. However, the wide concept of an obstetric near miss has delayed the adoption of a uniform definition and various criteria have been used in different studies: severity of illness, scoring of symptoms, grading of organ dysfunction and management-based classification. More recently WHO has suggested which criteria should be used to perform the baseline assessment and how to proceed with the situation analysis in order to implement interventions for improving health care. Wherever feasible, depending on local contextual factors, baseline assessment should include severe pregnancy-related complications and key interventions [3]. Admission to intensive care unit (ICU) should also be considered an effective indicator of serious morbidity [4-7].

Near miss cases may be used to assess quality of obstetric care and are a potentially useful starting point for audits for several reasons: larger numbers of cases permit more lessons to be learned from the management of cases who survive than auditing the management of those who died. Surviving women may be interviewed and risk factors ascertained, inappropriate care objectively documented as well as the patient’s own perception of the care she received.

The ratio of maternal deaths to maternal near miss cases can be calculated and compared between regions or over time, provided definitions are clarified [3]. It may indicate an area where change could be implemented in order to address a deficiency in practice. It also permits the development of preventive and educational programs with improved allocation of resources in order to achieve a reduction of both maternal morbidity and mortality [7,8].

Several studies on maternal morbidity and mortality have been published in sub-Saharan Africa over the last decade [9-12]. In Mozambique, Maputo Central Hospital (HCM) is the source of most published studies and clinical audit has come to be standard practice in “the Maputo model” [11-13]. Data from Maputo Central Hospital Maternity in 2004, showed that 777 patients were admitted in HCM ICU, 362 (46.6%) due to hypertensive disease, 170 (21.9%) with hemorrhagic complications and 56 (7.2%) with sepsis. Other contributing indirect causes mainly comprised malaria and HIV/AIDS (Bique Osman, personal communication). In this country one of the most prominent challenges is to reduce maternal mortality, recently estimated at 599 maternal deaths per 100,000 live births [14].

The purpose of this study was to assess the prevalence of near miss cases and maternal deaths in Maputo City and Province in southern Mozambique and analyzing avoidable factors associated with various delays in getting appropriate care. By better identifying circumstances surrounding severe morbidity and maternal deaths, this study aimed at improving essential obstetric care services and at providing relevant information to policy makers and planners to more effectively target interventions apt to reduce maternal morbidity and mortality.

## Methods

A cross-sectional study with continuous enrolment of study participants from August to December 2008, covering Maputo City (urban area) with a population of 1,094,315 (Census 2007) [15] and Maputo Province (rural area) with a population of 1,205,709 [16]. Data collection was carried out in five health facilities offering comprehensive emergency obstetric care (CEmOC), specifically Maputo Central Hospital (HCM), Mavalane General Hospital, José Macamo General Hospital, Manhiça District Health Centre and Xinavane Rural Hospital. These five public health units receive critically ill patients referred from all peripheral health units within the public national health system located in Maputo province. HCM is the biggest public hospital, the only one with an ICU for obstetric cases, a neonatal intensive care unit, and a team of obstetricians and anesthesiologists available round the clock. HCM provides quaternary care and serves as the major referral centre for other public and private hospitals to a region of approximately 1.2 million inhabitants, including about 360,000 women of reproductive age in the municipality of Maputo (Census 2007) [15].

Two groups of patients were enrolled using an exhaustive sampling approach. Firstly, pregnant women admitted in the labor ward or in the gynecology emergency ward, surviving the near miss event. Secondly, pregnant or puerperal women suffering a maternal death.

Near miss cases were identified in the five above mentioned health facilities according to the clinical criteria presented in Table 1.

**Table 1:** **Clinical criteria for identification of near-miss cases**

Eclampsia	Convulsions during pregnancy or in the first 48 hours postpartum together with hypertension (≥140/90 mmHg) and proteinuria (1+ on random dipstick analysis or 300 mg in 24 hours)
Severe hemorrhage	Profuse vaginal bleeding with hypovolemic shock, systolic blood pressure <90 mm Hg and need of blood transfusion. (cases of third trimester hemorrhage, post-partum hemorrhage and abortion)
Severe sepsis [17]	Two or more of the following signs: temperature >38°C or <36°C, heart rate >100 beats/minute, respiratory rate >20/min, white cell count >17×10^9^/l, clinical signs of peritonitis
Uterine rupture	Acute dehiscence of the uterus, no matter the mode of delivery, with need of blood transfusion and/or surgical repair and/or hysterectomy
Severe malaria [18]	Malaria with coma or convulsions and need of blood transfusion in the pregnant, puerperal or post-abortion women, were include

The following definitions were used:

- Maternal mortality ratio (MMR): maternal deaths per 100,000 live births.
- Case fatality rate: number of deaths due to a specific morbidity divided by the total number of diseased (survivors and deaths) with the same morbidity.
- Maternal Near Miss Ratio (MNMR): number of near miss cases per 1,000 live births.

### Data collection

Information was collected from the medical records. To complete information gaps in patient’s files and to facilitate the audit in near miss cases, an interview was conducted with the patient and accompanying family members, health workers or any other relevant person involved in her care using a pre-coded and partly open-ended questionnaire. Information included socio-economic background, reproductive characteristics, maternal and neonatal outcome. Also, efforts to seek care, accessibility of health services, management and problems encountered during management were recorded for the qualitative analysis.

Efforts were made to address the interviewees in an empathetic manner, avoiding blame and assuring confidentiality. Whenever necessary the interview was conducted in the patient’s local dialect.

In some instances of maternal death, in the absence of information coming from the deceased woman, members of the research team visited the family and conducted a home interview after two weeks from the event. A verbal autopsy was conducted aiming to uncover circumstances, clinical signs/symptoms and socio-economic factors contributing to the death.

Interviews were used to reconstruct the pathway and the series of events that might have contributed to the occurrence of near miss cases.

The approach was based on the conceptual framework of the three phases of delay in reaching/receiving care, according to Thaddeus and Maine definitions [19]:

First delay is related to community level, the decision to seek an appropriate obstetric emergency care and is influenced by the actors involved in decision-making; this delay is due to e.g. sociocultural factors, distance from the health facility, financial and opportunity costs.

Second delay is related to the difficulty to reach an appropriate obstetric facility, which depends on how far away the nearest facility is from home in terms of distance and travel time, availability and cost of transportation and road conditions.

Third delay is related to inadequate care when a facility is reached: factors affecting the receipt and provision of care (includes the adequacy of the referral system); shortages of supplies, equipment, trained personnel and lack of competence of available personnel.

Each case was discussed with all members of the study team together with a senior researcher and interviews were analyzed using a qualitative content analysis approach [20]. Facts/circumstances related to the near miss event, as they were described by the woman or the family, were evaluated for their contributing role to anyone of the 3 delays. It was judged as appropriate that more than one delay could be found for the same case. The text was carefully read to identify the meaning units. Key words and phrases were condensed. The principal investigator and co-investigators contributed to the interpretation of the underlying meaning. Coding was then done and codes assigned to the fitting type of delay (out of the 3 delays).

Filling of the questionnaire was completed soon after admission in most cases, while in the remaining before discharge. This resulted in almost no loss of information.

### Laboratory tests

HIV test was performed for each case, whenever possible.

### Ethical considerations

Near miss cases and their families, as well as the family of the deceased woman, were interviewed after informed consent regarding the study and its objectives.

This study was approved by the Mozambican National Bioethical Commission.

## Results

During the study period, there were 27,916 live births, 564 near miss cases and 71 maternal deaths. This resulted in a maternal near miss ratio of 20,2 per 1,000 live births and maternal mortality ratio of 254 per 100,000 live births. The aggregate case fatality rate was 11.2% (71/635). Hemorrhage accounted for the most common event (58.0%) and included 110 ectopic pregnancies, 59 miscarriages complicated with severe bleeding, 160 cases of 3^rd^ trimester hemorrhagic complications (placental abruption, placenta previa, postpartum hemorrhage).

Eclampsia occurred in 35.5% of cases, followed by infection (3.9%), uterine rupture (2.3%) and cerebral malaria (0.4%) (Table 2).

**Table 2:** **Characteristics and frequencies of near miss cases and maternal deaths at hospital level in Maputo city and Maputo province, Mozambique**

	**Total**	**Hemorrhage**	**Eclampsia**	**Sepsis**	**Uterine rupture**	**Cerebral malaria**
Live births	27,916					
Maternal near-miss (MNM)	564	327 (58.0%)	200 (35.5%)	22 (3.9%)	13 (2.3%)	2 (0.4%)
Maternal deaths (MD)^*^	71	27 (38.0%)	9 (12.7%)	4 (5.6%)	1 (1.4%)	2 (2.8%)
MMR	254	96.7	32.6	14.3	3.6	7.2
MNMR	20					
SMOR	22.7					
Case fatality rate^**^ (%)	11.2	7.6	4.3	15.4	7.1	50

MMR: maternal deaths per 100,000 livebirths.

SMOR: severe maternal outcome ratio (MNM + MD)/ 1000LB.

MNMR: severe morbidity per 1000 live births.

^*^To complete the total of 71 maternal death cases there are other causes of maternal death as AIDS 23 (32.4%), various 5 (7.0%).

^**^Maternal deaths devided by the sum of maternal deaths and near-miss cases.

Most near miss cases (70.7%) were referred to Maputo Central Hospital from peripheral health facilities, with a path that included the smaller health centers and the general hospitals mentioned in the Methods section. Accordingly only 29.3% of women came straight from home.

Socio-demographic characteristics of the near miss and maternal deaths groups are presented in Table 3. It is noteworthy that 23.6% of near miss patients were adolescents (14–19 years of age), 33.2% were not in a stable marital condition and 46.3% came from suburban areas.

**Table 3:** **Socio-demographic and reproductive characteristics of near-misses and maternal deaths**

	**Near miss cases**	**Maternal deaths**
	**n = 564 (%)**	**n = 71 (%)**
Age (yy)		
14-19	133 (23.6)	6 (8.5)
20-24	152 (27.0)	16 (22.5)
25-29	148 (26.2)	20 (28.2)
30-34	94 (16.7)	22 (31.0)
≥35	37 (6.6)	3 (4.2)
Missing	-	4
Range	14 – 47	16 – 42
Mean ± SD	25.0 ± 6.2	27.0 ± 5.2
Education		
None	39 (6.9)	5 (7.0)
Primary (7 years)	221 (39.2)	24 (33.8)
Secondary (12 years)	294 (52.1)	17 (23.9)
Higher (>12 years)	10 (1.8)	1 (1.4)
Missing	-	24
Marital status		
Single	187 (33.2)	13 (18.3)
Married/Cohabiting	367 (65.1)	34 (47.9)
Divorced	5 (0.9)	-
Missing	-	24
Residency		
Urban	60 (10.6)	1 (1.4)
Suburban	261 (46.3)	49 (69.0)
Rural	242 (42.2)	12 (16.9)
Missing	1	9
Parity		
0	191 (33.9)	6 (8.5)
1	117 (20.7)	15 (21.1)
2 – 4	229 (40.6)	32 (45.1)
≥ 5	27 (4.8)	6 (8.5)
Previous C-section		
1	35 (6.2)	6 (8.5)
2	13 (2.3)	2 (2.8)
3	9 (1.6)	-
Gestational age in the 1st antenatal control (trimester)		
I	54 (14.3)	3 (4.2)
II	246 (65.1)	29 (40.8)
III	78 (20.6)	7 (9.9)

One third of near miss cases (33.7%) had no antenatal control in the index pregnancy; among women who had gone through antenatal care, 85.7% had their 1^st^ antenatal visit after the first trimester (Table 3). According to the patient’s antenatal card, when available, 13.6% of women had been considered by staff to have a high risk pregnancy, and 28.0% had suffered from complications in previous pregnancies or deliveries such as stillbirth, ectopic pregnancy, ante partum hemorrhage, placental retention, postpartum hemorrhage or miscarriage.

**Table 4:** **Relevant information related to delay in decision, transport and care among near miss cases**

**Factor**	**n = 564 (%)**	
Decision to seek care		
Patient	165 (29.3.)	
Husband/partner	147 (26.1.)	
Relatives	215 (38.1)	
Neighbours	21 (3.8)	
Transport		
Owner car	257 (45.6)	
Public transport “Chapa”	173 (30.7)	
On foot	114 (20.2)	
Other	9 (1.6)	
Place of birth*		
Home	8 (2.1)	
Along the way	9 (2.4)	
Peripheral health unit	83 (21.7)	
Referral center	285 (73.8)	
Mode of delivery^*^		
Vaginal	167 (43.4)	
Caesarean	218 (56.6)	
Assistance at birth^*^		
Nurse/midwife	132 (34.5)	
Obstetrician/surg. technician	260 (60.5)	
Assistant medical officer	3 (0.8)	
Traditional birth attendant	1 (0.3)	
Relative	12 (3.1)	
Others	3 (0.8)	

^*^Near-miss cases associated with fetal viability (N = 382).

Among the near miss cases that reached a gestational age of fetal viability, mode of delivery was vaginal in 43.4%, mostly attended by midwives. Home delivery had occurred in 2.1% of cases (Table 4). A traditional doctor had been consulted by the woman or relatives in 9.4% (53/564); few women (n = 49) admitted having used a traditional remedy.

**Table 5:** **Contributing factors according to the 3-delays method (for near-miss cases)**

Factors associated with delay in seeking health care (1^st^ delay)	n = 360 (63.8%)
Desire for home delivery (traditional orientation, lack of confidence in the health system, etc.)	
Lack of information/knowledge of the problem	
Inadequate antenatal care (late attendance, delayed visits, etc.)	
Non-compliance with health provider’s advice (medication intake, referral to appropriate care, etc.)	
Belief in alternative care (relying on traditional healers or natural remedies)	
Refusal of treatment for an unwanted pregnancy (circumstancial evidence of induced abortion)	
Factors associated with delay in reaching the health system (2^nd^ delay)	n = 120 (21.3%)
Lack of resources (money, transportation means, roads,etc.)	
Distance	
Factors associated with delays within the health system (3^rd^ delay)	n = 393 (69.7%)
Delay in patient admission, patient referral, patient treatment	
Lack of resources (blood derivates, operating theatre, ultrasound, etc.)	
Sub-standard care (inappropriate diagnosis/treatment,unfriendly attitude,untrained health worker,etc.)	

HIV seroprevalence was 22.3% for near miss cases. Among maternal deaths it was possible to draw blood in a limited sample of 46 cases, out of which 35 (76.1%) were seropositive.

Neonatal outcomes of near miss cases were collected in 385 cases: 115 were stillbirths (29.9%); among the 270 newborns, 15 died within the 1^st^ week, giving a total perinatal mortality of 33.8% (130/385). Women who went through more than one health facility while seeking appropriate care, contributed to 67.7% of feto-neonatal loss, whereas the adolescent group (14–19 years old) suffered a 14.6% of adverse perinatal outcome.

Information on neonatal outcome related to maternal deaths was available in only 36 out of 71 women: in this smaller sample, which represents half of the whole group, 19 deaths were recorded at birth or within the 1^st^ week, giving a perinatal death rate of 52.7% (19/36).

### Interviews with patients and relatives

Contributing factors to any delay in seeking care are presented in Table 5, together with frequencies and sub-categories of 1^st^, 2^nd^ and 3^rd^ delays for near miss cases. There was no loss of information between quantitative and qualitative data. More than one delay was found in 363/564 (64.4%) cases.

The 1^st^ type of delay occurred in 63.8% of all cases due to various attitudes related to the woman’s traditional beliefs, perception of institutional health care services, etc. Reportedly, the decision to seek care was taken by the woman herself in 29.3% of the near miss cases, while in the remaining cases the woman depended on the husband’s or other family members’ decision.

A significant 2^nd^ type of delay could be found in 21.3% of cases due to lack of resources and the difficulty in covering long distances to reach health facilities.

The 3^rd^ type of delay, was identified in 69.7% of the interviews: delays in transfer and treatment together with various forms of substandard care such as lack of blood derivates (42.0%) and unavailable operating room (35.0%). More than one fourth of near misses reported no initial treatment within the first 30 minutes after arrival.

Only 10% of near misses stated that they were satisfied with the quality of care provided by the health services but few (6.0%) actually complained, blaming the health unit for lack of responsibility. When women with a near miss event were asked to comment on their condition, almost half of them answered that they did not know what had happened or if the life-threatening condition could have been prevented.

## Discussion

This is the first prospective report of maternal near miss cases and deaths in health facilities at provincial level in Mozambique. The study shows that severe acute maternal morbidities frequently affected women managed in the facilities investigated. Life-threatening obstetric conditions, with outcome either in near miss or in maternal death, complicated up to 2.3% of total livebirths. Our estimates of total maternal near-miss and maternal mortality ratio fall within the wide range of ratios reported in studies from other low-income countries, which used similar criteria of near-miss definition, based on severe maternal complications [21-23]. Observed maternal near miss prevalence of 20.2 per 1000 livebirths is similar to what was seen in the maternal near miss study done in a Sudanese rural hospital (22.1‰) [21], and also to the findings (21.2‰) of another surveillance study carried out in Brazil [23]. A recent systematic review estimated the prevalence in African countries ranging between 6 and 150‰ [24]. Our results concerning maternal deaths compare favourably with the findings of the Sudanese study, which reported 432 deaths/100,000 live births [21].

Over all, hemorrhage and eclampsia were predominant causes and contributed to more than 90% of our severe maternal morbidities, a much higher figure than the findings of the Sagamu study (61.6%) [22]. Comparing the mortality index of the two studies, we found lower figures for hemorrhage and eclampsia, likely due to the availability of blood bank services and the introduction of magnesium sulfate for severe pre-eclampsia management in almost all health facilities.

More than two thirds (70.7%) of near miss cases were referred from other health facilities, implying deterioration of the woman and her newborn’s condition due to longer time in transfer of the patient between facilities and delayed adequate care.

In the present study we observed a very low degree of antenatal attendance among the near miss population: 9 out of 10 had no first trimester visit and 1/3 of near miss cases had absolutely no antenatal control in the index pregnancy before the near miss event. Although it remains to be demonstrated that antenatal care leads to improved survival [25], we believe that the educational role of antenatal care can be considered a relevant factor in the reduction of maternal morbidity and mortality.

The attitude observed in the study group profoundly differs from that of the general Mozambican pregnant population, estimated to have a high antenatal attendance rate - 92.4% getting at least one visit during pregnancy [26]. The issue of reduced antenatal care includes also the competence of identifying the high risk pregnancies: our results suggest that proper identification of pre-existing risk factors also needs improvement. This is reflected by the discrepancy found between the proportion of women who had suffered an obstetric complication in a previous pregnancy in contrast with the prevalence of women recognized as having a high risk pregnancy, according to current national criteria.

Pregnancy among adolescent girls is a common situation in Mozambique. A study done in Maputo City in 2001 showed that 19.0% of a large sample of adolescents referred they had been pregnant at least once [27], but according to a 2011 enquiry, this figure has increased to 38% [28]. Our findings of a rather high prevalence of adolescents among near miss cases (23.6%) may indicate that this group is at high risk of adverse obstetric outcome in Mozambique. Compared with a Brazilian study [23], which showed a 14% prevalence of pregnancy among adolescents, our results have worse implications for this group of young, uneducated and family dependant girls, with lack of awareness of danger signs and limited decision making power. The figure reveals that this group is highly exposed to severe maternal morbidity as mortality.

Among maternal deaths, AIDS had a high contribution: 23/71 (32%) is similar to the findings in a Burkina Faso study (33.3%) [29], which correlates with the HIV seroprevalence of 22% found among near miss cases; this figure is higher than the background estimate of 16% affecting the pregnant population in Maputo province [30-32]. These publications emphasize that the HIV-infection prevalence is higher in the center and south of the country and in the female population.

Using the three delays framework [19] the qualitative data showed that 63.8% of the cases were related to the first delay. This figure is extremely high compared to the 14.5% reported by Amaral et al. [23] but does not differ substantially with regards to the findings of a Liberian study (38.0%) nor with the experience of Hirose et al. (54.0%) in Afghanistan [33,34].

The most common factors contributing to the first delay were related to culture (lack of empowerment, lack of information, lack of knowledge of the problem). Delivering at home is still viewed as a normal option and this perception is well proven by the 2008 statistics which indicate a 58% national coverage of institutional deliveries in Mozambique [35].

The second delay was not as frequent as the other two types of delay, although various precarious means of transportation were used and as much as 20.2% of women reached the closest health facility on foot. In spite of our low prevalence of 21.3%, there are studies that have reported a 3-fold prevalence [34], emphasizing the importance of distance from health facilities as well as lack of money for transportation when evaluating “what went wrong” along the path to medical care. However, according to the Authors of the Afghani study, most of the patients did not acknowledge the importance of this contributing factor. This attitude could also explain the low prevalence of the 2^nd^ delay found in our near miss sample, due to the fact that sometimes people accept as “normal” a current situation, no matter it’s implications, such as facing chronic lack of transportation.

On the basis of our results, a third delay was identified and frequently recurred in the interviews of the patients and family members. Delay might have started at first admission, continued within the referral system and in some cases was worsened by the lack of blood derivates or operating theatre.

Another delay at the facility level related to substandard care and to the attitude of the health workers towards the woman. Only a minority of our cases were satisfied with the quality of care provided by the institution, even though very few (6%) actually complained and placed blame on the health unit.

According to a published overview of the 5^th^ Uganda Demographic and Health survey, 2012, the third delay “calls on the moral imperatives and ethical responses from health care workers to prioritize the care afforded to women”, which would have “major implication for health policies, funding and leadership to ensure a reduction in the interval between onset of a complication and its efficient management in all health facilities” [36].

## Conclusion

Prospective monitoring of five health facilities in Maputo city and province and evaluation of the circumstances surrounding near miss cases, resulted in the finding of a high proportion of preventable, serious maternal complications. In doing so, this study has the potential to raise the awareness of the type of maternal complications which may evolve into a maternal death. Medical and non-medical factors have been explored, resulting in the definition of multiple delays, which hamper the process of seeking and receiving care.

On the basis of the results of this study it can be inferred that the woman and her family members dangerously postpone the decision to seek care. Due to hesitation and poor transport the woman eventually reaches the health facility in a badly worsened condition, often to face a last and fatal institutional delay.

The implementation of community based interventions in order to educate families, women and health workers may result in early problem recognition and prompt referral. Criteria based audit of surviving near miss mothers could become an integral part of health facility practices to improve quality of obstetric care.

Sexual and reproductive health services for younger women should be prioritized.

### Study limitations

Laboratory constraints were the reason to use the clinical approach to define the inclusion criteria. A limitation of this study therefore may be the exclusion of the management indicators in the process of identifying severe cases. The third delay and the evaluation of the care given could have been improved by including clinical audit both at the peripheral and central level.
